# Stem Cells and Gene Therapy for Cartilage Repair

**DOI:** 10.1155/2012/168385

**Published:** 2012-02-16

**Authors:** Umile Giuseppe Longo, Stefano Petrillo, Edoardo Franceschetti, Alessandra Berton, Nicola Maffulli, Vincenzo Denaro

**Affiliations:** ^1^Department of Orthopaedic and Trauma Surgery, Campus Bio-Medico University, Via Alvaro del Portillo 200, Trigoria, 00128 Rome, Italy; ^2^Centro Integrato di Ricerca (CIR), Università Campus Bio-Medico, Via Alvaro del Portillo, 21, 00128, Rome, Italy; ^3^Centre for Sports and Exercise Medicine, Barts and The London School of Medicine and Dentistry, Mile End Hospital, 275 Bancroft Road, London E1 4DG, UK

## Abstract

Cartilage defects represent a common problem in orthopaedic practice. Predisposing factors include traumas, inflammatory conditions, and biomechanics alterations. Conservative management of cartilage defects often fails, and patients with this lesions may need surgical intervention. Several treatment strategies have been proposed, although only surgery has been proved to be predictably effective. Usually, in focal cartilage defects without a stable fibrocartilaginous repair tissue formed, surgeons try to promote a natural fibrocartilaginous response by using marrow stimulating techniques, such as microfracture, abrasion arthroplasty, and Pridie drilling, with the aim of reducing swelling and pain and improving joint function of the patients. These procedures have demonstrated to be clinically useful and are usually considered as first-line treatment for focal cartilage defects. However, fibrocartilage presents inferior mechanical and biochemical properties compared to normal hyaline articular cartilage, characterized by poor organization, significant amounts of collagen type I, and an increased susceptibility to injury, which ultimately leads to premature osteoarthritis (OA). Therefore, the aim of future therapeutic strategies for articular cartilage regeneration is to obtain a hyaline-like cartilage repair tissue by transplantation of tissues or cells. Further studies are required to clarify the role of gene therapy and mesenchimal stem cells for management of cartilage lesions.

## 1. Introduction

Hyaline articular cartilage is a highly specialized tissue. The function of cartilage is to protect the bones of diarthrodial joints from friction, forces associated with load bearing and impact [1, 2]. The peculiar problem of this tissue is its durability. Once articular cartilage is injured or degenerated, it has very limited capacities for self-repair and regeneration. In partial thickness lesions, in whom the defect is completely contained within the articular cartilage, there is no involvement of the vasculature. Consequently, chondroprogenitor cells from marrow or blood cannot reach the damaged region to repair the lesion or contribute to the healing of the tissue. The most considerable consequence of cartilage avascularity is that articular chondrocytes are not able to migrate towards the lesion and to produce reparative matrix to fill the defect. As such, the defect is not repaired and remains permanently [1, 2].

Full thickness cartilage lesions result in the damage of the chondral layer and subchondral bone plate. The rupture of blood vessels promotes the formation of the hematoma at the injury site. In this condition, the repair response is promoted and the defect is filled with fibrocartilaginous tissue within weeks [1, 2].

Usually, in focal cartilage defects without a stable fibrocartilaginous repair tissue formed, surgeons try to promote a natural fibrocartilaginous response by using marrow stimulating techniques, such as microfracture, abrasion arthroplasty, and Pridie drilling with the aim of reducing swelling and pain and improving joint function of the patients. These procedures have demonstrated to be clinically useful and are usually considered as first-line treatment for focal cartilage defects [3–5]. 

However, fibrocartilage presents inferior mechanical and biochemical properties compared to normal hyaline articular cartilage, characterized by poor organization, significant amounts of collagen type I, and an increased susceptibility to injury, which ultimately leads to premature osteoarthritis (OA).

Therefore, as outlined in the modern literature on the subject, the aim of future therapeutic strategies for articular cartilage regeneration is to obtain a hyaline-like cartilage repair tissue by transplantation of tissues or cells [2, 3, 6–8].

Tissue transplantation procedures such as periosteum, perichondrium, or osteochondral grafts have shown positive results for a limited number of patients, especially in the short term, but long-term clinical results are uncertain, with tissue availability for transplant that seems to be the major limitation, especially in large cartilage defects [2, 3, 6–8]. The autologous chondrocyte transplantation (ACT) procedure has been performed since 1987 in combination with a periosteal cover to treat chondral or osteochondral lesions of the knee, reporting good clinical results [9–11].

Recently, several authors improved this procedure embedding chondrocytes in a three-dimensional matrix before transplantation into cartilage defects [4, 12, 13].

Good results have also been obtained especially regarding clinical symptoms, such as pain relief and joint motion, but none of the current treatment options has proved the capacity to reproduce the biochemical properties of articular hyaline cartilage [3, 10, 14].

Moreover, in the last years, tissue engineering approaches have been investigated with the aim to produce cartilage grafts *in vitro *to facilitate regeneration of articular cartilage *in vivo*. While promising *in vitro *data have been obtained compared to current cartilage repair options, various problems remain unresolved for a successful repair associated with the formation of hyaline cartilage *in vivo* [2, 7, 15, 16].

## 2. Gene Therapy

The gene transfer to articular tissues was firstly described and performed by Evans et al., as a method to treat patient with rheumatoid arthritis [17, 18]. Initial successful experiments in several animal models using retroviral-mediated gene delivery promoted subsequent clinical trials to evaluate the safety and feasibility of using gene therapy for rheumatoid arthritis [17, 18]. The study was performed on 9 patients without any complications; all the nine participants tolerated the treatment and, in addition, in all the treated joints, intra-articular gene transfer and expression was observed [17, 18]. The relative success of these studies suggests that this new treatment option can be used in major articular disorders for which only unsatisfactory treatment options are currently available.

Nowadays research and recent results indicate that the design of a successful genetic treatment for cartilage repair and restoration includes a refined strategy of gene delivery that takes into account the complexities of treating this particular tissue.

For the purpose of cartilage repair, potentially useful complementary DNAs (cDNAs) include members of the transforming growth factor- (TGF-) *β* superfamily, including TGF-*β*s 1, 2, and 3, a number of bone morphogenetic proteins (BMPs), insulin-like growth factor- (IGF-) 1, fibroblast growth factors (FGFs), and epidermal growth factor (EGF).

Alternatively, to support production and maintenance of the proper hyaline cartilage matrix, delivery, and expression of cDNAs encoding specific extracellular matrix (ECM) components such as collagen type II, tenascin, or cartilage oligomeric matrix protein (COMP) may also be used [19].

Another class of biologics that may be useful in cartilage repair is represented by transcription factors that promote chondrogenesis or the maintenance of the chondrocyte phenotype. SOX9 and related transcription factors (i.e., LSOX5) and SOX6 have been identified as essential for chondrocyte differentiation and cartilage formation [20].

Signal transduction molecules, such as SMADs, are also known to be important regulators of chondrogenesis [21]. However, since these molecules function completely in the intracellular environment, gene transfer may represent the only way to harness these factors for repair, as they cannot be delivered in soluble form.

Other secreted proteins, such as indian hedgehog (IHH) or sonic hedgehog (SHH), play key roles in regulating chondrocyte hypertrophy [22] and could be beneficial for modulating the chondrocytic phenotype of grafted cells.

Prevention or treatment of cartilage loss may also require the inhibition of the activity of certain proinflammatory cytokines, such as interleukin- (IL-) 1 and tumor necrosis factor- (TNF-) *α*, as these are important mediators of cartilage matrix degradation and apoptosis after trauma and disease. Therefore, anti-inflammatory or immunmodulatory mediators, such as interleukin-1 receptor antagonist (IL-1Ra), soluble receptors for TNF (sTNFR) or IL-1 (sIL-1R), IL-4 or IL-10, inhibitors of matrix metalloproteinases, and others, may be administered to effectively reduce loss of repair cells and matrix [23].

Inhibitors of apoptosis or senescence, such as Bcl-2, Bcl-XL, hTERT, i(NOS) and others, may also be beneficially employed to maintain cell populations which are capable of favourable repair responses at the injury site [24, 25]. Different candidate cDNAs may also be administered in combination, especially when favouring complementary therapeutic responses. For example, the combined administration of an anabolic growth factor (e.g., IGF-1) together with an inhibitor of the catabolic action of inflammatory cytokines (i.e., IL-1Ra) has the potential both to control the matrix degradation and to allow partial restoration of the damaged cartilage matrix [26, 27].

There are two general modes of intra-articular gene delivery, a direct *in vivo* and an indirect *ex vivo *approach. The direct *in vivo *approach involves the application of the vector directly into the joint space, whereas the *ex vivo *approach involves the genetic modification of cells outside the body, followed by retransplantation of the modified cells into the body.

The choice of which gene transfer method as to be used depends on several considerations, including the gene to be delivered, and the vector used. In general, *in vivo *and *ex vivo* delivery can be performed using adenovirus, herpes simplex virus, adenoassociated virus vectors, lentivirus, and nonviral vectors. Due to their inability to infect nondividing cells, retroviral vectors are more appropriate for *ex vivo *use. While *ex vivo *transfer methods are generally more invasive, expensive, and technically wearisome, they finally allow control of the transduced cells and safety testing prior to transplantation. *In vivo *approaches are simpler, cheaper, and less invasive, but these methods require the introduction of viruses directly into the body, which limits safety testing [28].

Towards the treatment of damaged articular cartilage, the three primary candidate cell types to target genetic modification are synovial lining cells, chondrocytes, and mesenchymal stem cells.

Direct intra-articular injection of a recombinant vector [29–31] represents the most straightforward strategy for gene delivery to diseased joints. Cartilage and synovium are the two primary tissues to be considered for this application.

Within articular cartilage, chondrocytes are present at a low density and are located at varying depths within the dense matrix. Due to this situation, it has not been possible to achieve an efficient genetic modification of chondrocytes *in situ* [32–35]. Conversely, gene delivery within the synovium tissue has resulted much more feasible since it is usually characterized by a thin lining of cells that covers all internal surfaces of the joint except that of cartilage. Also, because of its relatively large surface area, the synovium represents the predominant site of vector interaction. Both the implant of modified cells and direct intra-articular injection of vector promote the synthesis and release of therapeutic proteins into the joint space, which then bathe all available tissues, including cartilage.

Substantial progress has been made in defining the parameters that are critical for effective gene transfer to synovium and prolonged intra-articular expression by using different types of vectors in *ex vivo *and *in vivo *approaches. Through research conducted in the field of rheumatoid arthritis, the effectiveness of synovial gene transfer of various transgenes has been well documented [23]. *Ex vivo *gene delivery to joints has been taken into phase I clinical trial and shown to be feasible and safe in humans with rheumatoid arthritis [17, 36]. Data relevant to direct intra-articular gene delivery are beginning to emerge, although to date most of the work in this field has been focused towards the study and treatment of rheumatoid arthritis, mainly because of the potential of this approach in treating OA [37], and also to expand repair methods of focal cartilage defects [28, 38–40].

For example, encouraging results have been reported for adenovirally delivered IGF-1 or IL-1Ra using animal models for OA and localized cartilage injury [32, 41].

Through both direct and *ex vivo *gene transfer to synovium, it is possible to obtain biologically considerable levels of transgene expression while for delivery of certain growth factors, this approach is not compatible. In fact, it was observed that adenoviral mediated delivery of TGF-*β*1 or BMP-2 to the synovial lining determined osteophytes, cartilage degeneration, joint fibrosis, and significant swelling [42–45]. In the perspective of cartilage repair, these results suggest that synovial gene transfer may be more appropriate for the delivery of chondroprotective agents rather than strong anabolic transgenes with pleiotropic effects of their products. It has been shown that this property is common to many anti-inflammatory cytokines.

For the gene-based delivery of certain intracellular proteins or growth factors, it appears that a strategy based on increased localization of the transgenes with the gene products contained in the lesion of the cartilage may be more practical. To achieve this goal, the most direct approach may be represented by implantation into a defect of a three-dimensional matrix preloaded with a gene delivery vehicle, allowing infiltrating cells to acquire the vector and secrete the stimulating transgene products locally [37, 46].

In order to increase the healing of ligaments and bones, cartilage implants, activated genetically, have been designed [47–52]. For example, it has been seen that hydrated collagen-glycosaminoglycan matrices containing adenoviral vectors stimulate localized reporter gene expression *in vivo *for at least 21 days, after implantation into osteochondral defects localized in rabbit knees [50].

However, it is not known yet if this type of approach can promote an adequate biological response for repair due to the limited cell supply commonly present at the site of the cartilage lesion. To increase the graft cellularity, while preserving the feasibility of the procedure within one operative setting, autologous cells which are intraoperatively readily available, such as cells from bone marrow aspirates, could be mixed together with the genetically activated matrix. This genetically enhanced approach for tissue engineering would allow both the reduction of costs and execution time, while avoiding a significant effort for the *ex vivo *culture of cells [49, 50]. Nevertheless, the lack of control over gene transfer following implantation represents a limitation for their use.

Through the use of genetically modified chondrocytes, attempts have been made to further improve the quality of repaired tissue. Although chondrocytes have shown a certain resistance to transfection with plasmid DNA, it has been observed that some lipid-based formulations increase the efficiency of DNA uptake [53]. However, viral-based vectors are capable of producing far higher levels of transgene expression with enhanced persistence. It was found that transfection of monolayer-expanded chondrocytes with viral vectors such as Moloney Murine Leukemia Virus (MLV), lentivirus, adenovirus, and AAV occurs promptly. It has also been shown that adenoviral-mediated delivery of various transgenes, such as TGF-*β*1, BMP-2, IGF-1, or BMP-7, stimulates the production of a cartilage-specific matrix rich in proteoglycans and collagen type II and reduces tendency towards dedifferentiation [54–58].

It has been seen that following transfer of cDNA encoding matrix molecules, such as the collagen type II minigene, an increased extracellular matrix production occurs in human fetal chondrocytes [37].

Collagen type II expression of chondrocytes in three-dimensional culture *in vitro* has shown to be increased following transduction with the transcription factor SOX-9 [59, 60], whereas overexpression of the transcription factor Runx-2 (Cbfa-1) promotes chondrocyte maturation and determines a hypertrophic phenotype, expressing high levels of collagen types II and X, alkaline phosphatase, and osteogenic marker genes [61, 62].

Since it has been found that chondrocyte biology can be positively influenced by genetic modification, attention of research has focused on their efficient delivery to cartilage defects. The delivery of genetically modified chondrocytes in suspension has represented the first approach. Several studies demonstrated that after engraftment onto cartilage explants *in vitro,* genetically modified chondrocytes have the ability of expressing transgene products at functional levels [63].

Compared to transplanted control cells, in these systems, genetic modification with IGF-1 [64], FGF-2 [65], or SOX9 [66] resulted in a considerable resurfacing and thicker tissue containing increased levels of proteoglycans and collagen type II [53]. Moreover, adenoviral-mediated IL-1Ra gene transfer to chondrocytes led to resistance to IL-1-induced proteoglycan degradation after engraftment [67].

Genetically modified chondrocytes have also been used as an alternative to delivery in suspension with the aim of enhancing tissue engineering procedures. This approach requires the transduction/transfection in monolayer cells subsequently seeded into a matrix for further transplantation into chondral or osteochondral lesions. Several transgenes including TGF *β*1, BMP-2, -4, -7, IGF-1, SOX9, among others have shown promising results in these three-dimensional culture systems due to their ability to maintain and stimulate the chondrogenic phenotype *in vitro* [16, 28, 40].

Initial studies highlighted that following genetic modifications with adenoviral, AAV, retroviral, or plasmid vectors, chondrocytes had the ability to efficiently express reporter genes in chondral and osteochondral lesions, and that when the genetically modified chondrocytes were seeded in three-dimensional matrices, transgene expression was extended over several weeks [68–71].

The results of efficacy studies demonstrating the effects of genetically modified chondrocytes in cartilage defects *in vivo* have just started to be reported.

In an *ex vivo *approach, adenovirally transduced chondrocytes expressing BMP-7 [54], integrated in a matrix of autogenous fibrin, were implanted into full thickness articular cartilage lesions in horses [54]. An enhanced tissue volume with increased production of a proteoglycan and collagen type II rich matrix was detected 4 weeks after surgery in the BMP-7-treated lesions, compared to control lesions treated with unrelated marker genes.

After 8 months, the mechanical features of the treated lesions as well as the levels of collagen type II and proteoglycan were however similar compared to the controls. This finding was attributed to some extent to the reduction of the number of allografted chondrocytes that persisted after 8 months in the lesions [54]. Nevertheless, these findings remain encouraging since they suggest that genetically modified chondrocytes can be used to increase a cartilage repair process in a large animal model.

## 3. Mesenchymal Stem Cells

Until recently, scientists have mainly focused on research involving two types of stem cells from humans and animals: nonembryonic “somatic” or “adult” stem cells and embryonic stem cells.

Embryonic stem cells are present in the blastocyst while adult stem cells are found in adult tissues. The normal turnover of organs that have a high intrinsic regenerative ability which include blood, skin, and intestinal epithelium is maintained by adult stem cells. Adult stem cells are generally unipotent or multipotent and they can be found in adults as well as adolescents and children.

Adult pluripotent stem cells are normally found in small numbers since they are very rare. However, they are present in several tissues including umbilical cord blood. The adult stem cells studied most extensively to date are the multipotent stem cells which are commonly referred to by their tissue origin (i.e., hematopoietic stem cells that differentiate into platelets erythrocytes, white blood cells, etc.) and the bone marrow stromal cells (also known as MSCs) [72, 73], which have the capacity to differentiate into connective tissue cells.

MSCs have the potential to differentiate into cells of connective tissue lineages [74] including bone [75–77], cartilage [77–79], ligament [80–82], muscle [78], fat [78, 83], and IVD [81, 82, 84]. It has been detected that these cells are also capable of differentiation along myogenic and neurogenic lineages, although these are not the common pathways used to prove multipotentiality of isolated MSCs.

Originally, adult MSCs were isolated from bone marrow by Pittenger et al. in 1999 [74], who demonstrated the potential for multilineage differentiation of these cells. Subsequently, a number of studies allowed to demonstrate the presence of stem cells in various adult tissues, including synovial fluid, articular cartilage, synovial membrane, periosteum, dermis, muscle, and adipose tissue.

To date, research has allowed for MSC-like progenitor cells isolation from trabecular bone, periosteum, synovium, skeletal muscle, adipose tissue, deciduous teeth [78, 80], and bone marrow [85].

Since no definitive markers of MSCs are available, a range of cell surface markers are normally used. These include immunopositivity for STRO-1, CD73, CD105, CD106, CD145, and CD166, associated with negative immunoreactivity for CD11b, CD31, CD34, CD45, and CD117.

Compared to the previous methods based on either density-gradient centrifugation or even simple plastic adherence, these markers allow to identify a more homogeneous population of cells.

Due to general heterogeneity of bone marrow cell populations, variable results can be obtained; however, MSCs have commonly shown the ability to differentiate along the adipogenic, chondrogenic, and osteogenic pathways. Research conducted by several authors suggests that MSCs are capable of differentiation to chondrocytes, osteoblasts, and nucleus pulposus (NP) cells of the IVD [84, 86–88]. However, since no definitive markers of NP cells are available, a number of chondrocyte markers, with which they share a large phenotypic similarity, are typically used.

After Pittenger et al. [74] demonstrated the chondrogenic potential of MSCs, a number of approaches promoting MSC chondrogenesis [60] such as agarose [89] and alginate [90] gels have been described and more recently a range of tissue engineering biomaterials which allow or promote chondrogenesis have also been reported.

One of the most commonly used growth factors is TGF-b [74, 91], which has shown to promote chondrogenesis in addition to inhibiting adipogenic and osteogenic differentiation [92, 93].

Growth factors of the BMP family, principally BMP-7, and IGF-1 have also demonstrated the ability to promote chondrogenesis of MSCs and it has also been suggested that expansion of monolayer MSCs in medium containing FGF-2 induces chondrogenesis following transfer to a 3D culture environment [94–97].

However, with the *in vitro* differentiation approaches, the complexity of the signaling pathways involved in chondrogenesis represents one of the major problems, compared to the simplicity of culture systems.

Several studies have demonstrated the importance of cell-cell contact for MSC differentiation to either NP cells or chondrocytes [73] and pellet cultures mimic the mesenchymal compression that occurs during embryogenesis.

Similarly it is known that differentiation and matrix formation are induced by anabolic growth factors that exert their activity through a number of pathways, primarily the Smad and MAPKinase pathways [92, 96, 98].

The routine assessment of successful chondrogenesis is performed by the induction of SOX-9, which subsequently promotes the production of type II collagen as well as the enhanced expression of the PG aggrecan [99, 100]. Based on the similarities in the phenotype of NP cells of the IVD and articular chondrocytes [101], these markers are also used routinely to identify NP-like cells since no validated and highly specific NP marker genes are available. However, in standard *in vitro* culture systems MSC differentiation has shown to be unstable and it commonly leads to the expression of hypertrophic markers such as alkaline phosphatase and type X collagen [91, 102].

In terms of clinical application, the likelihood that chondrogenic differentiation may cause hypertrophy represents a problem since healthy surface and mid zone chondrocytes and NP cells do not express alkaline phosphatase nor type X collagen [103, 104].

This was demonstrated by Pelttari et al. [105] in pellet cultures comparing MSCs and chondrocytes, who reported that following implantation into SCID mice, the MSCs showed high levels of alkaline phosphatase and type X collagen expression which induced vascular invasion and calcification, while chondrocytes produced a cartilaginous matrix.

Improved differentiation or terminal differentiation inhibition may be induced with a number of growth factors. For example, it has been observed that the addition of PTHrP to TGF-b3-stimulated MSCs in poly-glycolic acid scaffolds also inhibits the expression of type X collagen of these cells and suppresses their terminal differentiation [106]. Also, the combination of TGF-b3 with BMP-2 has shown improved chondrogenic differentiation of MSCs compared to either growth factor alone or the combination of TGF-b3 with either BMP-4 or BMP-6 [107].

## 4. Conclusions

Hyaline articular cartilage is a highly specialized tissue. The peculiar problem of this tissue is that once articular cartilage is injured or degenerated, it has very limited capacities for self-repair and regeneration.

Usually, in focal cartilage defects without a stable fibrocartilaginous repair tissue formed, surgeons try to promote a natural fibrocartilaginous response by using marrow stimulating techniques, such as microfracture, abrasion arthroplasty, and Pridie drilling [108–111].

However, fibrocartilage presents inferior mechanical and biochemical properties compared to normal hyaline articular cartilage, characterized by poor organization, significant amounts of collagen type I, and an increased susceptibility to injury, which ultimately leads to premature OA [112–114].

The implementation of gene transfer techniques may allow to overcome the limitations of the current treatments for articular cartilage lesions. It has been shown that various approaches could be appropriate for an efficient transfer of exogenous cDNAs to cartilage lesions *in vivo *and for achieving sustained expression of the related gene products.

Initial efficacy studies have proven that gene-transfer techniques represent potent tools able to promote a significant biological response *in vivo*. However, the safety of gene transfer approaches for cartilage repair is also of particular importance because cartilage injuries are not life-threatening. Therefore the application of this technology for clinical use is strongly dependent on the use of safe and efficient delivery systems vectors and transgenes.

Although a number of animal models for OA and other types of arthritis are available, none of them allow to predict the equivalent disease in humans and most them are linked with problems. Further studies are required to establish the role of stem cells and gene therapy for cartilage repair.
